# A Novel Step Length Estimator Based on Foot-Mounted MEMS Sensors

**DOI:** 10.3390/s18124447

**Published:** 2018-12-15

**Authors:** Zhuangsheng Zhu, Shibo Wang

**Affiliations:** School of Instrumentation and Optoelectronic Engineering, Beihang University, Beijing 100191, China; zszhu@buaa.edu.cn

**Keywords:** pedestrian navigation, step length, inertial navigation, human motion

## Abstract

Pedestrian Dead Reckoning (PDR)-based pedestrian navigation technology is an important part of indoor and outdoor seamless positioning services. To improve the performance of PDR, we have conducted research on a step length estimator. Firstly, based on the basic theory of inertial navigation, we analyze in detail the errors in traditional Strapdown Inertial Navigation Systems (SINSs) caused by the unique motion state of pedestrians. Then, according to the fact that the inertial data from the foot can directly reflect the gait characteristics, we conduct a step length estimator that does not rely on SINS. The experimental results show that accuracy of the proposed method is between 0.6% and 1.4% with a standard deviation of 0.25%.

## 1. Introduction

With the arrival of the intelligent and information era, different forms of pedestrian navigation technology, such as Wireless Local Area Network (WLAN) positioning [1], Ultra-wide Bandwidth (UWB) positioning [2], Global Navigation Satellite System (GNSS) [3], Locata system and GPSOne positioning are gradually infiltrating into all aspects of social life to meet the needs of people’s fast-paced life, and improving people’s quality of life significantly. However, these positioning technologies require a signal source that is susceptible to interference and occlusion, so their application environment or application range has certain limitations. More importantly, these positioning technologies can’t be used in the complex and volatile emergency rescue environment.

Inertial navigation is a classic and very vital navigation method. It can work independently without any external electrical, magnetic, acoustic, optical and other information, and is not interfered by any signal [4]. In addition, due to the development of microelectromechanical systems (MEMS) technology, inertial sensors have become smaller, lighter, and cheaper [5], which provides the possibility to achieve navigation positioning only through wearable inertial devices. Since the 1990s, PDR technology based on inertial measurement has attracted the attention and research of many researchers [6], which has led the inertial sensor to truly serve indoor and outdoor seamless positioning. PDR systems can be divided into two categories [7]: the first is the Strapdown Inertial Navigation Systems (SINS) method, and the second is the Step-and-Heading System (SHS) method. The SINS method for pedestrian navigation is all derived from traditional vehicle and aircraft navigation. However, the “non-rigid” characteristics of pedestrians and the local motion characteristics of a certain part of the body do not satisfy the theoretical basis of traditional SINS. Therefore, the SINS method faces certain limitations when it comes to pedestrian navigation. Compared with the SINS method, the SHS method converts the position error accumulated over time into the position error accumulated with the step count at the cost of reducing the frequency of navigation information, which greatly reduces the speed of error accumulation. Therefore, the SHS method is a relatively reliable way for pedestrian navigation.

SINS is a classic navigation system, using the inertial data of the installation point and combining the rigid body rotation theory, the navigation information of the carrier can be given in real time. However, for pedestrian navigation based on SINS, the points used for mounting sensors on the human body (such as the foot or waist) have local motion, and this motion is not enough to express the motion state of the whole body. The above phenomenon will introduce SINS cone error and MEMS gyroscope G-sensitivity error: The cone error is due to the non-commutability of the rigid body rotation [8,9,10], which is amplified by the rate of angular velocity caused by the “non-rigid” and local motion characteristics of the pedestrian; The G-sensitivity error is an intrinsic property of micromechanical devices [11,12], which is amplified by the high motion acceleration caused by the local motion characteristics of pedestrians; a detailed analysis of the above two kinds of errors is provided in Appendix A.1 and Appendix A.2 respectively. Meanwhile, due to the significant error of the MEMS sensors in pedestrian navigation, SINS position errors will quickly accumulate over time. To compensate the SINS error, zero velocity update (ZUPT) can be used [13] and it originates from the idea of applying parking time to correct the SINS error for the ground vehicle navigation. However, during normal walking, there is almost no installation point in a completely static state. Even the zero-speed state of the pedestrian’s foot is only an approximate stage [14]. In addition, the ZUPT algorithm can be affected by many factors, such as the stiffness of the mounting point or the accuracy of zero-speed detection, and it is also difficult to estimate the heading error [15] or position error [16] introduced by the movement characteristics of pedestrian effectively. In summary, the traditional SINS method will face certain limitations on pedestrian navigation. Therefore, this paper focuses on the research of the SHS method.

SHS is another pedestrian navigation method based on inertial measurement that mainly has three sub-algorithms: step detection [17]; step-length estimation [18]; heading estimation [19]. Although the heading estimation is the sub-algorithm that most restricts the final positioning accuracy, the step-length estimation is also a basic component of the SHS method and it is essential for the development of a wearable pedestrian positioning system. The step length estimators can be mainly divided into three categories: the first is the direct methods based on the geometric features of limb movement; the second is indirect methods based on the empirical step-length model; the third are methods based on optical or acoustic sensors.

The first method to measure step length defines the analytical expression for the step length based on the geometric relationship between some angles and displacements of different parts on the body. Some scholars [20,21] believe that the leg is in an inverted pendulum state during walking, and the analytical relationship between the step length and the vertical displacement of the pedestrian was proposed. Do’s [22], based on the inverted pendulum model, introduced the vertical upward displacement and vertical downward displacement of the waist into the step length estimator. Miyazaki [23] estimated the stride length using the leg length and the opening angle during the stride measured by a 1-axis gyroscope mounted on the thigh. Miyazaki found that the error in the estimation of this model depends on the walking speed. However, these methods need to integrate the inertial signal to achieve the displacement or angle, which inevitably faces drift errors.

For the step length estimator based on the empirical step-length model, there are various proposals. By training large amounts of data to find variables related to the step length, statistical regression methods [24,25] were used to construct a step length model. However, these methods must spend a lot of time on training and the limited sample data used in training will directly affect the accuracy and generality of the step length model. To reduce the complexity of the algorithm, Weinberg believed that the step length is related to the vertical acceleration and proposed a step length model based on the amplitude of the vertical acceleration during a step [26]. Kang [27] proposed an alternative expression that also considers the amplitude of the vertical acceleration during the step, looking for a better adaptation to different users. However, these methods usually require a calibration phase for adjusting their performance to different users. Meanwhile, more step-related physical quantities need to be considered for a better step length with higher precision.

For the step length estimator based on optical or acoustic sensor. Saarinen [28] used ultrasonic sensors mounted on the front and back of each shoe and can produce high quality displacement estimates. Based on optical flow method, Qian [29] uses the built-in cameras and inertial sensors of mobile phones to estimate pedestrian walking distance. However, these methods face low versatility caused by hardware complexity. In summary, the step length estimator has many suggestions, which are based on different models and assumptions, and are tested and evaluated in different ways. However, the step length estimator is still in the research stage and there is no gold standard method for it. This paper focuses on the construction of the step length model in the SHS method. Firstly, the feasibility of analyzing pedestrian gait characteristics based on angular velocity and acceleration of the foot is confirmed by real data. Then we analyze the problem caused by the special motion state of the pedestrian’s foot to the traditional SINS. Finally, a novel model-based step length estimator based on the foot-mounted MEMS sensor is proposed.

## 2. Analysis of Human Foot Motion Characteristics

**Figure 1:**
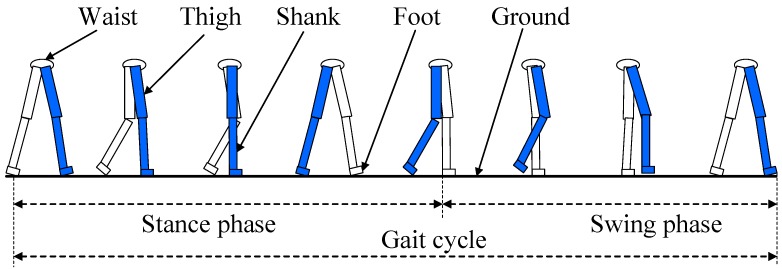
Division of the gait cycle.

Human walking is a cyclical and reciprocating movement. In the gait analysis of clinical medicine, the moment when the heel of the one-sided foot contacts the ground is usually selected as the start time of the gait cycle, and the moment when the heel is in contact with the ground again is selected as the end time of the gait cycle [30]. As shown in Figure 1, the gait cycle can be divided into a stance phase and a swing phase according to whether the foot is in contact with the ground [31].

In early studies, Yoo showed that linear motion or angular motion of human limbs can directly express the dynamic characteristics of gait, and these motions contain a large amount of information that can be used to study gait [32]. The following is a test to analyze the movement characteristics of pedestrians’ foot.

**Figure 2:**
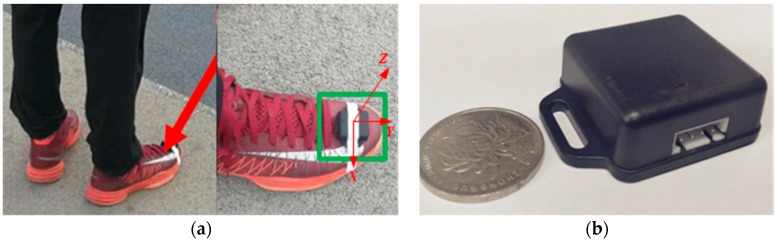
Applied equipment. (**a**) Installation method; (**b**) Exterior.

In this section, the MEMS sensor is mounted on a single foot. The installation mode is shown in Figure 2a. The coordinates of the sensor module are defined as follows: the Y-axis direction is along the toe direction; the Z-axis direction is perpendicular to the foot surface, and the X-axis conforms to the right-hand rule. Thus, the Y-axis accelerometer is sensitive to the front and rear motion of the foot; the Z-axis accelerometer is sensitive to the up and down motion of the foot; the X-axis accelerometer is sensitive to the left and right movement of the foot. The MEMS sensor used is the JY-901 module which integrates the MPU6050 chip (including a three-axis accelerometer and a three-axis gyroscope) and its external structure is shown in Figure 2b. The gyroscope detects the angular motion and the accelerometer detects the linear motion. The sensor’s performance parameters are listed in Table 1.

**Figure 3:**
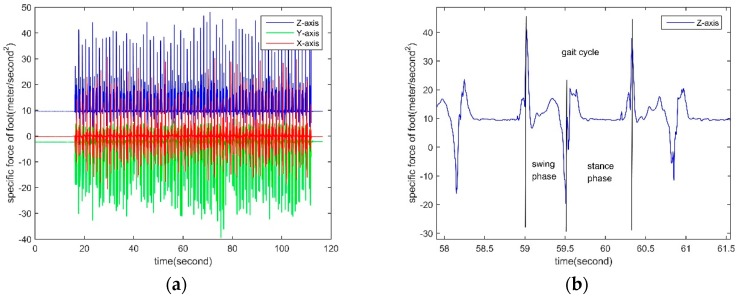
Specific force data of the foot. (**a**) Three-axis specific force data of the foot; (**b**) Partially enlarged for Z-axis specific force data of the foot.

The output information of the three-axis accelerometer on the foot during pedestrian walking is shown in Figure 3.

It can be seen from Figure 3a that the output information of the Z-axis accelerometer has good periodicity and reciprocity. Figure 3b is a partial enlarged view for the Z-axis accelerometer in Figure 3a. As can be seen from Figure 3b, the Z-axis accelerometer can determine the various stages experienced during a gait cycle during pedestrian walking.

**Figure 4:**
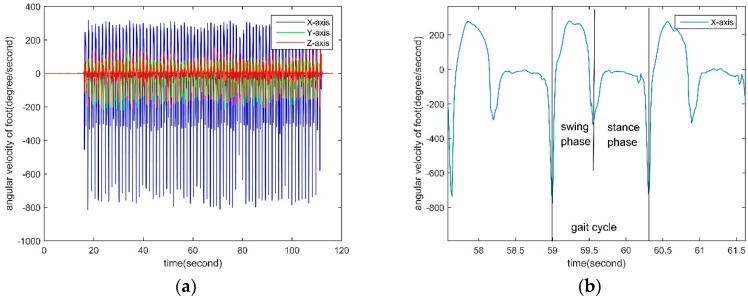
Angular velocity data of the foot. (**a**) Three-axis angular velocity data of the foot; (**b**) Partially enlarged for X-axis angular velocity data of the foot.

In the same way, the output information of the three-axis gyroscope on the foot during pedestrian walking is shown in Figure 4. It can be seen from Figure 4a that the output information of the X-axis gyroscope has good periodicity and reciprocity. Figure 4b is a partial enlarged for the X-axis gyroscope in Figure 4a. As can be seen from Figure 4b, the gait cycle can also be determined by the X-axis gyroscope.

In summary, for the inertial sensor mounted on the foot of the pedestrian, the linear motion information output by the Z-axis accelerometer or the angular motion information output by the X-axis gyroscope has obvious periodicity and reciprocity. This phenomenon can be used to analyze the gait characteristics during pedestrian walking.

## 3. Step Length Estimator Based on Foot-Mounted MEMS Sensor

**Figure 5:**
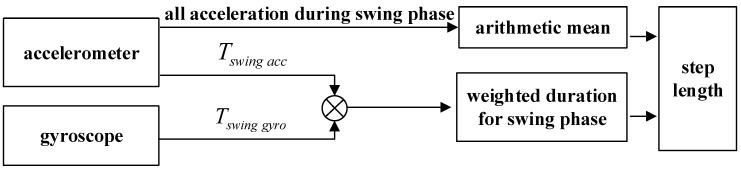
The flow chart of the proposed step length estimator.

The methods to measure step length involved in pedestrian navigation mainly include those based on the constant model [6], based on the empirical model [33] and based on the integral principle [34]. These methods each have certain problems: the step length estimator based on the constant model can’t meet the randomness of the actual human motion; the step length estimator based on the empirical model needs to further apply the parameters related to the step size, thereby improving the versatility and accuracy; the step length estimator based on the integral principle relies too much on the zero-speed detection algorithm, and it can’t avoid the SINS error introduced by the special motion state of the pedestrian foot. Therefore, to achieve the step length using only MEMS sensor, we propose a pedestrian step length estimator method with time information, based on the existing idea of step length estimator from empirical model. The method measures the duration of the swing phase in each gait cycle by accelerometer and gyroscope, and then combines the acceleration information during the swing phase to obtain the step length. The flow chart is shown in Figure 5.

The first step in estimating the step length is to measure the duration of the swing phase. Based on the analysis of Section 2, during the walking process, the laws of acceleration and angular velocity from foot can directly reflect the sway phase, so we apply the acceleration signal and angular velocity signal simultaneously to detect the sway section.

First, construct a calculation method for the duration of sway phase based on the acceleration from foot:

(1) $$T_{1}=\left\{ \begin{array}{l} \frac{da_{z}(t)}{dt}{|}_{t<T_{1}}>0\\ \frac{da_{z}(t)}{dt}{|}_{t=T_{1}}=0\\ \frac{da_{z}(t)}{dt}{|}_{t>T_{1}}<0 \end{array}\right.$$

(2) $$T_{2}=\left\{ \begin{array}{l} \frac{da_{z}(t)}{dt}{|}_{t<T_{2}}<0\\ \frac{da_{z}(t)}{dt}{|}_{t=T_{2}}=0\\ \frac{da_{z}(t)}{dt}{|}_{t>T_{2}}>0 \end{array}\right.$$

In the Equations (1) and (2) *a***_z_**(*t*) is the Z-axis output signal of the accelerometer located at the foot; *T*_1_ is the time at which *a***_z_**(*t*) reaches its maximum in one gait cycle, which indicates the start time of the swing phase; *T*_2_ is the time at which *a***_z_**(*t*) reaches its minimum in one gait cycle, which indicates the end time of the swing phase; *d* is integral operation; *t* is the time unit. After Equations (1) and (2), all maxima or minima of *a***_z_**(*t*) can be obtained. Due to fluctuations in *a***_z_**(*t*), there may be multiple maxima or minima in each gait cycle, but there are only two extreme points describing the start time and end time of swaying phase in a gait cycle. Therefore, constraints are set to choose expected point:

(3) $$L1=\left\{ \begin{array}{l} 1\;(a_{z}(T_{1})>th_{1})\&(a_{z}(T_{1})=\max(a_{z}(T_{1}-\tau_{1}:T_{1}+\tau_{1})))\\ 0 \end{array}\right.$$

(4) $$L2=\left\{ \begin{array}{l} 1\;(a_{z}(T_{2})<th_{2})\&(a_{z}(T_{2})=\min(a_{z}(T_{2}-\tau_{2}:T_{2}+\tau_{2})))\\ 0 \end{array}\right.$$

In Equations (3) and (4), *th*_1_ and *th*_2_ have the same units as acceleration and are the constraint threshold for *a***_z_**(*T*_1_) and *a***_z_**(*T*_2_), respectively. As shown in Figure 3b, the maximum value of the Z-axis acceleration from foot is usually higher than 2 g, and the minimum value of the acceleration from foot is usually less than −0.5 g. Therefore, the two constraints of *th*_1_ = 2 *g* and *th*_2_ = −0.5 *g* can be chosen to achieve the first-step screening of all acceleration extreme points. *τ*_1_ and *τ*_2_ are the time domain constraint thresholds for *a***_z_**(*T*_1_) and *a***_z_**(*T*_2_), respectively. *τ*_1_ is used to ensure that *a***_z_**(*T*_1_) is the maximum value in a gait cycle. *τ*_2_ is used to ensure that *a***_z_**(*T*_2_) is the minimum value in a gait cycle. Since the step length is generated once in each gait cycle, *τ*_1_ and *τ*_2_ should be less than half of the gait cycle to ensure that the compared points are in the same gait cycle. Through experiments it has been found that the gait cycle generally lasts 0.7~1.8 s, so to achieve the second-step screening of all acceleration extreme points, *τ*_1_ = *τ*_2_ = 0.2 s can be chosen. max() is the maximum function. min() is the minimum function. *L*1 and *L*2 are logical variables. When *L*1 and *L*2 are both logic 1, the accelerometer-based, formula for the duration of swaying phase is:

(5) $$T_{swing\;acc}=\left\{ \begin{array}{l} T_{2}-T_{1}\;(L1\&L2)\\ NULL\;\mathrm{otherwise} \end{array}\right.$$

where *T_swing_*acc is duration of swaying phase calculated from acceleration signal, *NULL* means no result.

Then, we construct a calculation method for the duration of the sway phase based on the angular velocity of the foot:

(6) $$T_{3}=\left\{ \begin{array}{l} \frac{d\omega_{x}(t)}{dt}{|}_{t<T_{3}}<0\\ \frac{d\omega_{x}(t)}{dt}{|}_{t=T_{3}}=0\\ \frac{d\omega_{x}(t)}{dt}{|}_{t>T_{3}}>0 \end{array}\right.$$

(7) $$T_{4}=\left\{ \begin{array}{l} \frac{d\omega_{x}(t)}{dt}{|}_{t<T_{4}}<0\\ \frac{d\omega_{x}(t)}{dt}{|}_{t=T_{4}}=0\\ \frac{d\omega_{x}(t)}{dt}{|}_{t>T_{4}}>0 \end{array}\right.$$

where *ω_x_*(*t*) is the X-axis output signal of the gyroscope located at the foot; *T*_3_ represents the starting moment of the swing phase; *T*_4_ represents the end time of the swing phase. After Equations (6) and (7), all minima of *ω_x_*(*t*) can be obtained. Like the process of processing acceleration signals, constraints are set to choose expected point:

(8) $$L3=\left\{ \begin{array}{l} 1\;(\omega_{x}(T_{3})<th_{3})\&(\omega_{x}(T_{3})=\min(\omega_{x}(T_{3}-\tau_{3}:T_{3}+\tau_{3})))\\ 0 \end{array}\right.$$

(9) $$L4=\left\{ \begin{array}{l} 1\;(\omega_{x}(T_{4})<th_{4})\&(\omega_{x}(T_{4})=\min(\omega_{x}(T_{4}-\tau_{4}:T_{4}+\tau_{4})))\\ 0 \end{array}\right.$$

In the Equations (8) and (9), *th*_3_ and *th*_4_ have the same units as angular velocity and are the constraint threshold for *ω_x_*(*T*_3_) and *ω_x_*(*T*_4_), respectively. As shown in Figure 4b, the minimum value of the X-axis angular velocity from foot is usually less than −200°/s. Therefore, the two constraints of *th*_3_ = −200°/s and *th*_4_ = −200°/s can be set to achieve the first-step screening of all angular velocity extreme points. *τ*_3_ and *τ*_4_ are the parameters in time domain for constraining *ω_x_*(*T*_3_) and *ω_x_*(*T*_4_), respectively. Like the settings of *τ*_1_ and *τ*_2_, *τ*_3_ = *τ*_4_ = 0.2 s can be chosen. *L*3 and *L*4 are logical variables. When *L*3 and *L*4 are both logic 1, gyroscope-based, formula for the duration of swaying phase is:

(10) $$T_{swing\;gyro}=\left\{ \begin{array}{l} T_{4}-T_{3}\;(L3\&L4)\\ NULL\;\mathrm{otherwise} \end{array}\right.$$

where *T_swing gyro_* is the duration of the swaying phase calculated from the angular velocity signal.

Finally, after obtaining the duration of the swing phase based on the accelerometer and the gyroscope, respectively. Then according to Figure 3b and Figure 4b, the duration of the swaying phase calculated from acceleration and angular velocity is almost equal. Their influence on the duration of the swaying phase is close. Therefore, the weighted result with equal weights is obtained and the duration of the swing phase *T_swing_* is:

(11) $$T_{swing}=0.5(T_{swing\;gyro}+T_{swing\;acc})$$

The second step in measuring the step length is to construct the model. By analyzing the relationship between stride, step period and the vertical acceleration from ankle, Kim [35] proposes the step length estimator based on vertical acceleration from ankle, which can be expressed as follows:

(12) $$SL=0.98\times \sqrt[{3}]{\frac{\sum_{1}^{N}\left|A_{k}\right|}{N}}$$

where *SL* is the short name of step length, *N* is the number of sampling points in one gait cycle, *A_k_* is the vertical acceleration from ankle. Although the application of this method does not require a prior calibration process, when the algorithm is applied to different individuals, its accuracy is degraded due to the lack of consideration about differences between individuals.

At the same time, as can be seen from Section 2, each gait cycle can be divided into a swing phase and a stance phase. In the stance phase, the foot is in contact with the ground, and the foot does not move relative to the ground. Therefore, step length from the foot only occurs in the swaying phase, or the swaying phase is an important period in which the step length will be generated. 

Based on Kim’s idea of introducing the average value of acceleration into the step length estimator, and also, according to fact that step length from the foot only occurs in the swaying phase, a step length estimator with the duration of the swing phase and average acceleration from foot during the swing phase as inputs is proposed:

(13) $$SL=\frac{\sum_{i=1}^{n}\sqrt{\left(a_{x}{\left(i\right)}^{2}+a_{y}{\left(i\right)}^{2}+a_{z}{\left(i\right)}^{2}\right)}}{n}\times T_{swing}^{}{}^{2}\times K$$

where *a_x_*(*i*), *a_y_*(*i*), *a_z_*(*i*) are the three-axis direct output signals of the accelerometer mounted on the foot in the swing phase; *n* represents the number of samples for the accelerometer within the swing phase; *K* is a parameter related to the height and weight of the individual pedestrian, which needs to be calibrated for different users. 

## 4. Experiments

To verify the application effect of the proposed step length estimator, multiple experiments were conducted. Accuracy of the proposed algorithm can be achieved by the difference between actual walking distance (*WD*) and the measured walking distance (*MD*), as shown in Equation (14). At the same time, based on the experimental results, the proposed method has been compared with the ZUPT + SINS [36], Kim [35] and Zhang [37].

(14) $$Accuracy=\frac{\left|WD-MD\right|}{WD}\times 100\%$$

In our walking experiments, to ensure the reliability of the experimental process, some pre-calibrated trajectories with known length are described by marker lines. The experimenters walk along pre-calibrated trajectories and keep the foot with sensors on the marker lines when it touched the ground, so the known length for the pre-calibrated trajectories can be used to obtain the actual walking distance. If one experimenter has walked from the start point to the vicinity of the end point, then, using a ruler with a resolution of 1 cm we can measure the distance between the landing point and the end point of the pre-calibrated trajectory. Then, based on the standard distance between the end point and the start point of pre-calibrated trajectory, the distance between the landing point and the start point can be derived, which is the actual walking distance.

### 4.1. Experimental Equipment and Site

The JY-901 described in Section 2 is selected for collecting data. The JY-901 not only integrates basic inertial sensors, also a Bluetooth wireless transmission module, so that it can send inertial data to a smartphone. Then, data from the JY-901 can be stored by the smartphone-based software. During the experiment, inertial sensors were installed on the foot, ankle, and waist to obtain the inertial data required by multiple step length estimators. The JY-901 module with wireless transmission facilitates data storage.

**Figure 6:**
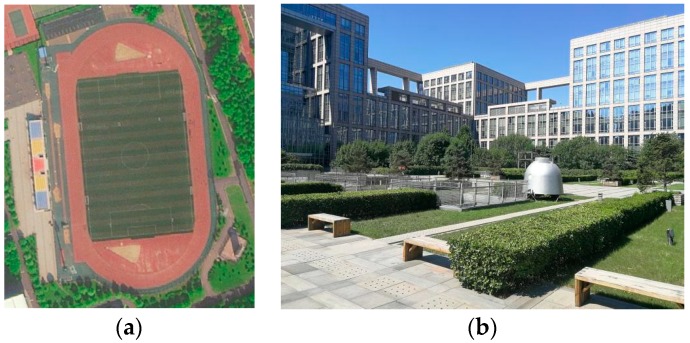
Experimental site. (**a**) The stadium of the Beihang Shahe Campus; (**b**) The central platform of the New Main Building of the Beihang Xueyuan Campus.

At the same time, we selected the stadium of the Beihang Shahe Campus and the central platform of the New Main Building of the campus as the experimental site. The experimental site is shown in Figure 6.

### 4.2. Experimental Participants and Their Calibration for Model Parameters

In order to verify the application effect of the proposed algorithm for different people, we selected ten students with different characteristics to participate in the walking experiments. 

Before performing experimental verification, the parameter *K* needs to be calibrated in advance. According to the Equation (13), when the step length, the duration of the swing phase and output signals of the accelerometer mounted on the foot during the swing phase are known, parameter *K* can be obtained by the Equation (15):

(15) $$K=SL/(T_{swing}^{}{}^{2})/(\frac{\sum_{i=1}^{n}\sqrt{\left(a_{x}{\left(i\right)}^{2}+a_{y}{\left(i\right)}^{2}+a_{z}{\left(i\right)}^{2}\right)}}{n})$$

**Figure 7:**
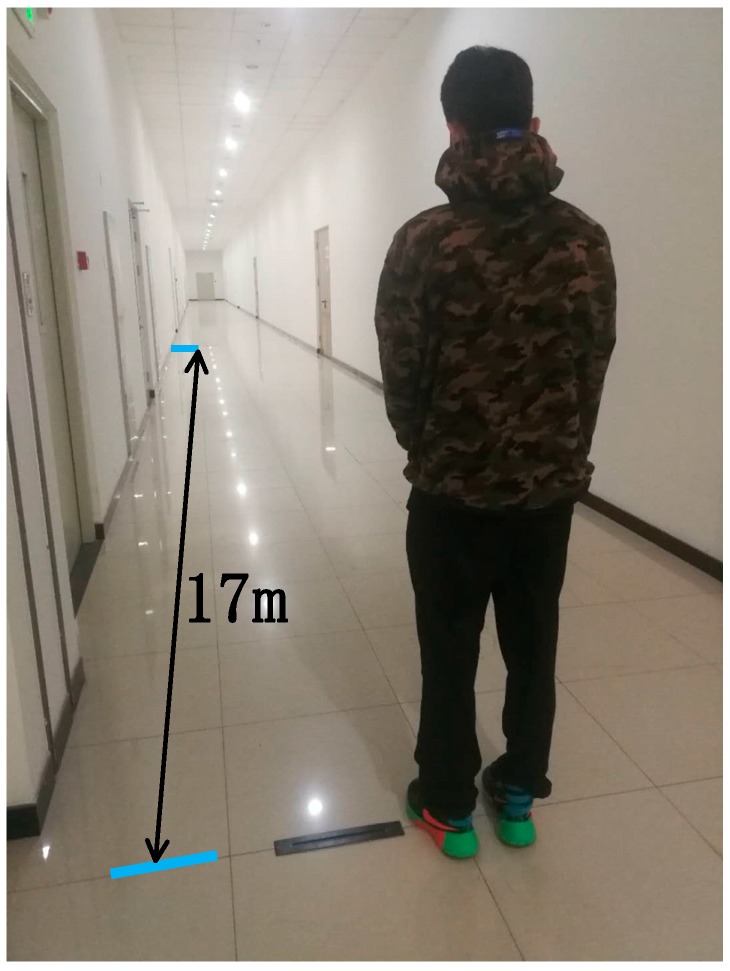
Parameter calibration process for student.

In order to obtain these above three necessary quantities for calibrating the parameter *K*, a linear space with a length of 17 m was selected for parameter calibration, and the ten students walked in this space with their usual gait with a foot-mounted sensor (Figure 7). 

The number of steps and the actual walking distance of each student were recorded to obtain an average step length. Inertial data from the foot for each step were recorded to obtain an average of the duration of the swing phase, also an average of output signals from the accelerometer during the swing phase. The calibration process and calibration results are given in Table 2.

### 4.3. The Walking Experiment with Complex Path

**Figure 8:**
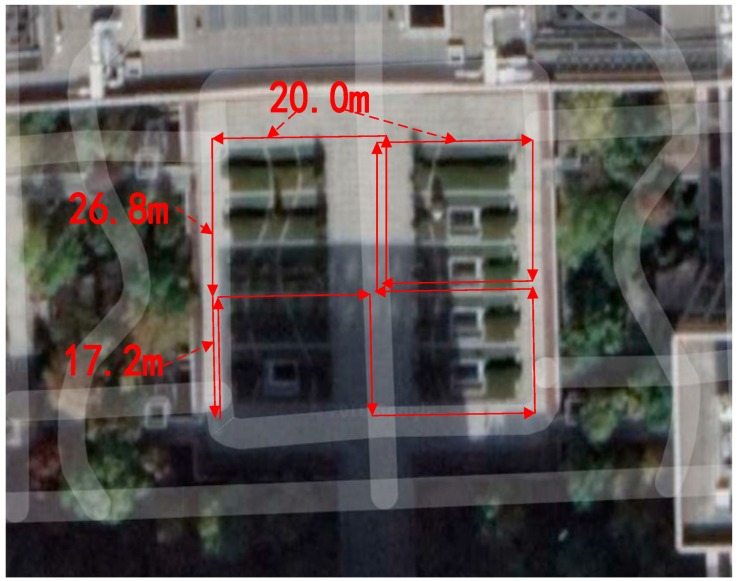
The reference trajectory.

To verify the application effect of the algorithm in the path where the direction changes multiple times, we selected a pre-calibrated trajectory on the central platform of the New Main Building as the reference trajectory, as shown in Figure 8. The reference trajectory contains a total of 1080° turns, with 630° to the right and 450° to the left, and a total distance of 296 m. During each experiment, each student walked along the pre-calibrated route. At the same time, in order to verify the adaptability of the algorithm to gait changes, the student changed pace about every four seconds during walking. The experimental results are shown in Table 3.

**Figure 9:**
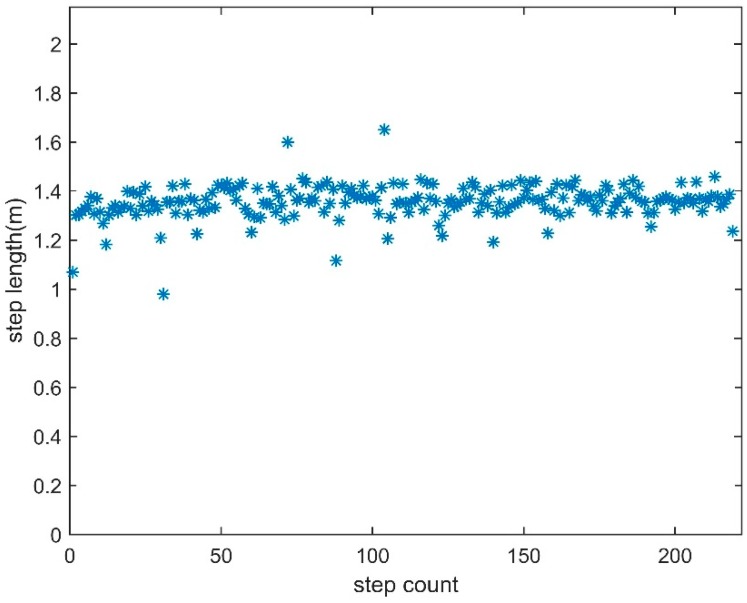
Experimental results for student 6.

We conducted some walking experiments with a total distance of about 2960 m. Based on the experimental results, the accuracy of the proposed step length estimator is between 0.8% and 1.3% with an average of 1.01%, which is superior to the traditional method. The experimenter usually needs to change the gait significantly at the inflection point of the path so that the foot can remain on the reference trajectory all the time, which currently causes a significant change in the step length. Therefore, in Figure 9, the measurement for step length at the inflection point of the path is generally different from other measured values. Moreover, during the walking process of the pedestrian, the pace changes about every four seconds do not significantly affect the accuracy of the proposed algorithm, although these pace changes have not reached the level of “walking to running”.

### 4.4. Long Distance Walking Experiment 

In order to verify the application effect of the proposed algorithm to long walking distances, the selected ten students have conducted long-distance walking experiments. During each experiment, each student walked a full 5 laps along the standard 400 m runway on the stadium of the Beihang Shahe Campus, so the actual walking distance of each student was 2000 m. At the same time, in order to verify the adaptability of the algorithm to gait changes, the students changed their pace about every twenty seconds during walking. The final experimental results are shown in Table 4.

We conducted some long distance walking experiments with a total distance of about 20,000 m. The accuracy of the step length estimator based on ZUPT + SINS is lower than the proposed method because pedestrians often change pace during the experiments, which reduces the accuracy of zero-speed detection. The step length estimator proposed by Kim has fixed model parameters that are easy to implement, but it ignores the differences between different users, which leads to lower precision (the average is 4.2%). The step length estimator proposed by Zhang need a sensor mounted at the waist to achieve indirect step length measurements, since the waist is not the power source of the step, the accuracy of this method fluctuates greatly (0.6~5.8%). The proposed step length estimator in this paper has relatively stable and reliable accuracy (0.6~1.4%).

### 4.5. Summary of Experimental Results

In summary, we conducted some walking experiments with a total distance of about 22,960 m. Experimental results show that accuracy of the proposed method is between 0.6% and 1.4% with a standard deviation of 0.25%.

## 5. Discussion

In most studies using Inertial Measurement Units (IMUs) mounted on the foot to measure walking distance [38], the ZUPT_SINS algorithm is heavily used. However, this method not only faces the error caused by failure of the ZUPT detection, but also cannot solve the additional error caused by the special motion state of the pedestrian’s foot. At the same time, we have to admit that the foot is the most direct part of human body to produce the pedestrian’s forward displacement, so the law of inertial data from the foot is a direct manifestation of the step length. Based on the above two points, we chose to measure the walking distance by mounting the sensor on the foot without SINS. Therefore, starting from a gait analysis, a novel step length estimator based on the foot-mounted IMU has been conducted, which can provide walking distance information. The proposed method uses the inertial data from the foot that most directly reflects the gait features to obtain the step length, so it is superior to the methods which measure the step length indirectly based on inertial data from other parts of the body (for example, the waist). Meanwhile, it does not perform the attitude update necessary for the traditional SINS, which can avoid the SINS cone error amplified by the pedestrian’s foot motion feature. Moreover, in the process of utilizing the angular velocity of the foot, the method acquires the gait feature only by the periodicity of the angular velocity instead of the accurate amplitude of angular velocity, which can avoid the G-sensitivity error amplified by the pedestrian’s foot motion feature. Therefore, the performance of this proposed method is also superior to the traditional SINS_ZUPT method.

Using a foot-mounted sensor for pedestrian navigation is always a contested point since it is not very suitable for everyday life. However, for emergency rescue areas that do not care too much about comfort or load capacity, it still has a broad application space. With the development of the manufacturing industry, shoes with embedded inertial sensors could also provide a wide range of applications for such methods. In this case, the calibration process for the parameters in the step length model will be limited. Therefore, further research is needed to identify the laws of the parameters to reduce the tediousness of the calibration process.

There are also some references that use a navigation method that mounts the sensor at the waist and provides distance measurements through a step model. However, the waist is not the direct initiator of the forward displacement for the pedestrian. Based on the inertial data from waist and the step length model, the walking distance can only be measured indirectly, which easily leads to inaccurate step length measurements. The waist movement is relatively stable, and the SINS installed at the waist faces a smaller attitude error caused by the pedestrian’s movement characteristics, so the waist is more suitable for providing heading information.

Different parts of the human body are suitable for providing different information. We believe that the future trend of inertial pedestrian positioning technology will be “based on multi-node sensor networks to achieve full utilization of pedestrian motion characteristics”.

Step length is only one of the important pieces of information to implement PDR. In the future work, we will design an effective technology to estimate heading for environment without satellite signals. Finally, based on the step length and heading, a complete PDR system will be conducted.

## Figures and Tables

**Table 1:** Performance parameters for JY-901.

Items	Accelerometer	Gyroscope
Range	±16 g	±2000°/s
Stability	0.01 g	0.05°/s
Data frequency	200 Hz	200 Hz

**Table 2:** Basic body characteristics and the calibration results of each student.

No.	Parameter *K*	Actual Distance (m)	Average Step Length (m)	Average *T_swing_* (s)	Average of Acceleration during the Swing Phase (g)	Height (m)	Weight (kg)	Gender
1	2.098	17.5	1.167	0.61	1.4949	1.52	48.7	Female
2	2.176	17.4	1.025	0.54	1.6154	1.54	46.9	Female
3	2.315	17.0	1.218	0.58	1.5640	1.60	50.2	Female
4	2.587	17.6	1.262	0.55	1.6126	1.68	55.2	Female
5	2.632	16.1	1.286	0.51	1.8785	1.68	62.1	Male
6	2.746	17.3	1.308	0.53	1.6957	1.78	57.6	Female
7	3.113	17.4	1.391	0.52	1.6524	1.79	70.4 kg	Male
8	3.465	17.2	1.436	0.55	1.3701	1.80	66.3 kg	Male
9	3.574	16.1	1.464	0.50	1.6385	1.84	71.6 kg	Male
10	3.692	17.3	1.597	0.49	1.8016	1.95	85.7 kg	Male

**Table 3:** Experimental results for the walking experiment with complex path.

No.	Actual Distance (m)	The Proposed Method		ZUPT + SINS [36]		Kim [35]		Zhang [37]	
		Measured Distance (m)	Error	Measured Distance	Error	Measured Distance (m)	Error	Measured Distance (m)	Error
1	296.5	299.7	1.1%	288.2	2.8%	283.2	4.5%	298.9	0.8%
2	296.3	293.6	0.9%	288.3	2.7%	284.7	3.9%	304.3	2.7%
3	295.4	292.7	0.9%	309.3	4.7%	307.8	4.2%	282.7	4.3%
4	296.1	298.5	0.8%	285.4	3.6%	285.1	3.7%	308.5	4.2%
5	296.8	300.7	1.3%	307.2	3.5%	311.3	4.9%	291.2	1.9%
6	296.5	292.9	1.2%	306.6	3.4%	313.1	5.6%	280.5	5.4%
7	295.7	293.6	0.7%	303.1	2.5%	309.6	4.7%	291.6	1.4%
8	296.9	293.0	1.3%	289.2	2.6%	286.2	3.6%	287.7	3.1%
9	295.6	298.0	0.8%	285.5	3.4%	303.9	2.8%	288.5	2.4%
10	295.6	298.9	1.1%	304.1	2.9%	286.1	3.2%	306.5	3.7%

**Table 4:** Experimental results for long distance walking experiment.

No	Actual Distance (m)	The Proposed Method		ZUPT + SINS [36]		Kim [35]		Zhang [37]	
		Measured Distance (m)	Error	Measured Distance (m)	Error	Measured Distance (m)	Error	Measured Distance (m)	Error
1	2000	1982.0	0.9%	2074.3	3.7%	2102.5	5.1%	2020.5	1.0%
2	2000	2016.3	0.8%	1948.1	2.6%	1916.3	4.2%	1936.6	3.2%
3	2000	2024.4	1.2%	1946.7	2.7%	2076.1	3.8%	2038.2	1.9%
4	2000	2012.3	0.6%	2070.0	3.5%	2092.0	4.6%	1908.8	4.6%
5	2000	1974.2	1.3%	2090.4	4.5%	1922.7	3.9%	2116.7	5.8%
6	2000	1984.4	0.8%	1942.5	2.9%	2082.6	4.1%	2072.0	3.6%
7	2000	2014.6	0.7%	2070.1	3.5%	1900.9	5.0%	2012.7	0.6%
8	2000	2022.0	1.1%	1946.9	2.7%	2084.2	4.2%	2040.5	2.0%
9	2000	1972.3	1.4%	2043.0	2.2%	1924.3	3.8%	1952.7	2.4%
10	2000	2026.7	1.3%	2056.9	2.8%	2058.0	2.9%	1976.8	1.2%

**Figure A1:**
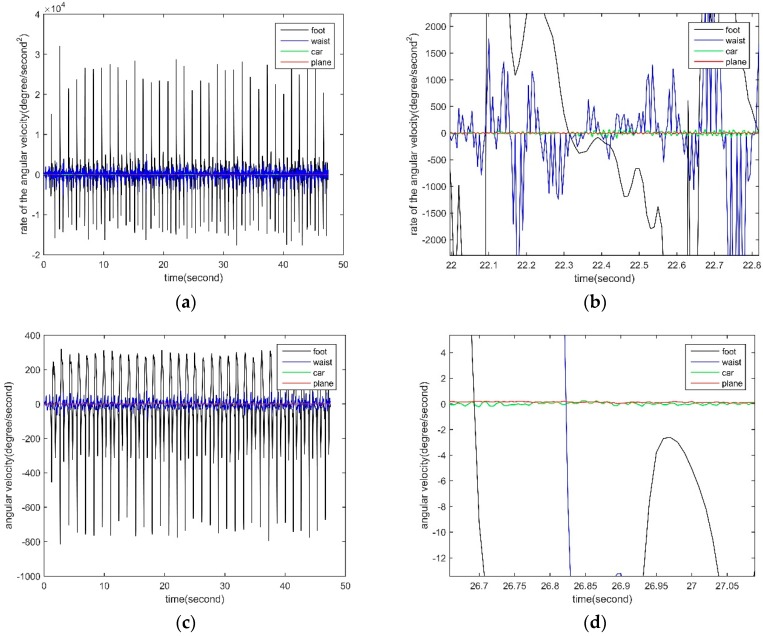
Angular motion data for different carriers. (**a**) Rate of angular velocity from four different carriers for car, plane, foot and waist; (**b**) Partial enlarged detail for (**a**); (**c**) Angular velocity from four different carriers for car, plane, foot and waist; (**d**) Partial enlarged detail for (**c**).

**Figure A2:**
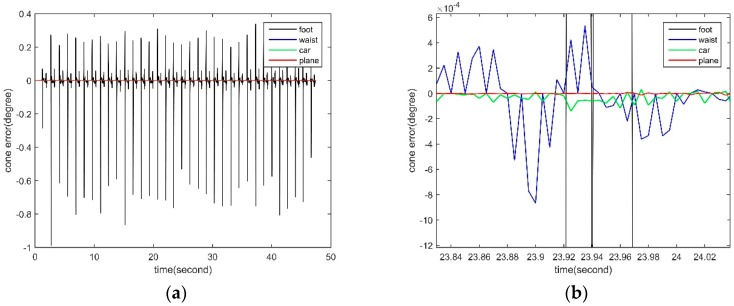
SINS cone error between each adjacent two sampling points. (**a**) SINS cone error between each adjacent two sampling points from four different carriers for plane, car, foot and waist; (**b**) Partial enlarged detail for (**a**).

**Figure A3:**
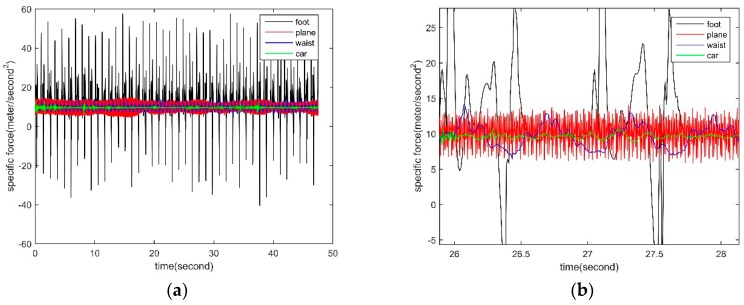
Line motion data for different carriers. (**a**) Specific force from four different carriers for car, plane, foot and waist; (**b**) Partial enlarged detail for (**a**).

**Figure A4:**
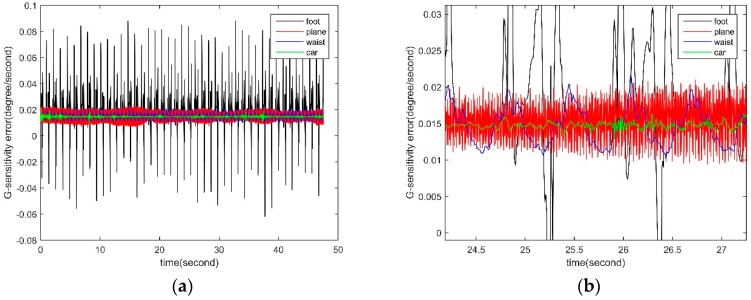
The G-sensitivity error corresponding to each sampling point. (**a**) The G-sensitivity error corresponding to each sampling point from four different carriers for plane, car, foot and waist; (**b**) Partial enlarged detail for (**a**).

## Appendix A

SINS can use the inertial information of the Inertial Measurement Unit (IMU) installation point and combine the rigid body rotation theory to give the navigation information of the carrier in real time. However, for SINS that serves pedestrian navigation, the physiological movement characteristics of pedestrians lead to significant local movements at the IMU installation site. Therefore, the method of using the local motion characteristics of the installation site to represent the entire human motion faces certain limitations. From the perspective of human physiology, during the walking process, various parts of the body have their own movements to maintain the coordination and stability of the body. For example, the vertical displacement of the pedestrian’s waist shows a state of approximate harmonic motion [39], the leg is like the movement of a single pendulum [22], and the foot is driven by the leg and rotates around the ankle [40]. From the perspective of pedestrian kinematics, the entire body of the pedestrian is often accompanied by movements with frequent changes in direction, frequent sudden stops or sudden movements. These special motion attributes of the human body will inevitably bring new errors to the traditional SINS. The following will focus on the error caused by the local motion of the mounting point to the traditional SINS.

### Appendix A.1. Cone Error Introduce by Pedestrian Movement

In rigid body mechanics, the finite rotation of a rigid body is not interchangeable, which determines that the rotation is not a vector, that is, two or more rotations cannot be directly added. Therefore, when the instantaneous angular velocity direction of the rigid body is constantly changing in space, there is a certain error in the process of integrating the angular velocity vector transformed in time in the space direction to obtain the change of the rotational angle [41]. which is:

(A1) $$\Delta \theta =A_{t}+\int_{t}^{t+\Delta h}\omega (t)dt$$

where *A_t_* is the calculation error (or non-exchangeable error) caused by the non-commutability of the rotation for the rigid body. *ω*(*t*) represents the angular velocity. Δ*θ* is the change in angle. Some scholars believe that when the SINS is performing attitude updates, the conical motion of the carrier will greatly amplify the non-exchangeable error that can’t be ignored and can induce serious drift to the digital platform, and this part of the error is called the cone error [42]. In order to suppress the cone error, Bortz proposed the concept of rotation vector [43], and gave the rotational vector differential equation to describe the rotation of the rigid body:

(A2) $$\begin{array}{l} \dot{\Phi }=\omega +\frac{1}{2}\Phi \times \omega +\frac{1}{\phi^{2}}[1-\frac{\phi \sin\phi }{2(1-\cos\phi )}]\Phi \times (\Phi \times \omega )\\ \phi =|\Phi | \end{array}$$

where Φ is rotation vector which can describe the attitude information of the carrier. |Φ| is the magnitude of the rotation vector Φ. Based on Taylor’s expansion, Equation (A2) can be simplified:

(A3) $$\dot{\Phi }=\omega +\frac{1}{2}\Phi \times \omega +\frac{1}{12}\Phi \times (\Phi \times \omega )$$

The latter two terms on the right side of the Equation (A3) can be regarded as an approximate analytical form of the cone error. It can be found that when the carrier rotation vector Φ has the same direction as the angular velocity *ω*, the cone error is almost zero. In the other words, when the angular velocity *ω* is not parallel to the current accumulated rotational vector direction Φ, the cone error is not zero. As shown in Figure 1, during walking, the foot will rotate around the ankle or knee or hip joint. These physiological characteristics can greatly increase the rate of angular velocity of the carrier (compared to conventional carriers such as cars, airplanes, ships, etc.), so that the angular velocity of the IMU mounted near the foot is frequently not parallel to the carrier rotation vector Φ. Finally increase the SINS cone error. Savage [44] gives a general analytical form of cone error corrections:

(A4) $$\delta con=\sum_{j=1}^{N-1}\sum_{i=j+1}^{N}C_{ij}\Delta \theta_{i}\times \Delta \theta_{j}$$

where *δcon* is the cone error corrections, *C_ij_* is the coefficient of SINS cone error corrections; Δ*θ_i_,* Δ*θ_j_* are the angular increments between two consecutive sampling points; *N* is the order of error correction, which is related to the number of sampling points during the attitude update cycle. If the carrier angular velocity remains the same, then:

(A5) $$\Delta \theta_{i}=\Delta \theta_{j}$$

According to the law of multiplication about two vectors, the corresponding cone error compensation correction is zero currently. Conversely, if the angular velocity of the carrier is changed, in other words, if the rate of angular velocity is not zero, then a cone error will be exhibited. We use the second-order correction of the SINS cone error derived by Savage [45] to quantitatively analyze this. The analytical form is as follows:

(A6) $$\left\{ \begin{array}{l} \frac{1}{12}\Delta \theta_{k-1}\times \Delta \theta_{k}\\ \Delta \theta_{k-1}=\int_{t_{k-2}}^{t_{k-1}}\omega dt\\ \Delta \theta_{k}=\int_{t_{k-1}}^{t_{k}}\omega dt \end{array}\right.$$

where *ω* is the angular velocity from the gyro output. To analyze the physical quantity that causes the SINS cone error, we can study from the mathematical analytical relationship. Assume that the varying angular velocity from the carrier causes Δ*θ_k−_*_1_ and Δ*θ_k_* to satisfy the following relation:

(A7) $$\Delta \theta_{k-1}=\left[\begin{array}{l} x\\ y\\ z \end{array}\right],\Delta \theta_{k}=\left[\begin{array}{l} x+\Delta x\\ y\\ z \end{array}\right]$$

where *x, y,* z represent the angular increments in three directions, Δ*x* indicates the angular increment caused by the changing angular velocity and it is proportional to the rate of angular velocity. Then the correction for cone error is:

(A8) $$\frac{1}{12}\left[\begin{array}{l} x\\ y\\ z \end{array}\right]\times \left[\begin{array}{l} x+\Delta x\\ y\\ z \end{array}\right]=\frac{1}{12}\left[\begin{array}{l} 0\\ z\Delta x\\ -y\Delta x \end{array}\right]$$

From Equation (A8), the correction for SINS cone error is proportional to Δ*x*, which is proportional to the change rate of angular velocity. Therefore, the SINS cone error is proportional to the rate of angular velocity, so the influence of the motion state from different navigation objects on the SINS cone error can be analyzed by the rate of angular velocity.

The angular velocity from different carriers during the smooth motion phase is collected, including the vehicle’s angular velocity, the aircraft’s angular velocity, the pedestrian’s waist angular velocity, and the pedestrian’s foot angular velocity. The above-mentioned rate of angular velocity data and its local amplification results are shown in Figure A1. Obviously, compared to the angular velocity of traditional object, such as an aircraft or car, the pedestrian’s angular velocity has a large rate of change, which increases the Δ*x* in Equation (A8) and then increases the SINS cone error.

According to the data in Figure A1, combined with Equation (A8), the cone error between each adjacent two sampling points can be obtained, as shown in Figure A2. The magnitude of the cone error caused by the movement of the foot is much larger than that of waist, vehicle and plane with relatively stable motion, and its amplitude is between −1° and +0.4°. To further quantitatively analyze the problem, the conic error in each gait cycle is numerically accumulated, and it can be found that the cumulative amount of conical error caused by the motion characteristics of the foot in each gait cycle is at least 0.8°. Considering that the pedestrian’s gait cycle is generally between 0.7 s and 1.3 s, it is concluded that the magnitude of the cone error caused by the pedestrian’s foot motion feature even exceeds the drift of the consumer MEMS gyro.

For the SINS cone error compensation, based on the data of the sampling point, Lee [8] fits the information of the non-sampling phase to a quartic polynomial and proposes a classic four-sub-sample rotation vector algorithm. Wang [9] studied high-order cone errors in a pure cone environment and proposed an optimized three-sample rotation vector method. The existing cone error compensation methods are mainly based on a polynomial fitting method to optimize the coefficient *C_ij_* and the order *N* in Equation (A4). However, the angular velocity of pedestrians has the characteristics of high rate of change, which leads to the traditional method for fitting becoming difficult to accurately describe the pedestrian’s angular velocity. Furthermore, for the process of determining the coefficient *C_ij_* in Equation (A4), a coefficient optimization scheme and an error approximation scheme according to the actual application environment need to be formulated [10]. However, there is currently no conic error correction scheme for pedestrian motion conditions. Therefore, when SINS is applied to pedestrian navigation, it is faced with the limitation of the coning error being magnified.

### Appendix A.2. Gyroscope G-Sensitivity Error Introduced by Pedestrian Movement

The output of acceleration is specific force, and the specific force is the vector difference between motion acceleration and gravitational acceleration. Therefore, the high motion acceleration can increase the specific force. For carriers such as vehicles, missiles, and planes, its acceleration is a gradual process. During the pedestrian movement, the body is often influenced by an external factor, such as point of interest or another person, which makes the pedestrian stop or start suddenly. Pedestrians’ feet also alternately perform a touch action and an instant start action. For these motion characteristics, the acceleration is a sudden change process, which leads to high specific force characteristics. The high specific force characteristics can be sensitive to the gyroscope installed on the human body, and finally amplify the G-sensitivity error of the gyroscope. The G-sensitivity error is [46]:

(17) δω=Gf

where *f* represents the specific force, and *G* represents the coefficient of G-sensitivity, *δω* is G-sensitivity error. Because the G-sensitivity error is proportional to *f*, the specific force caused by the motion of the carrier can be used to analyze the G-sensitivity error.

Accelerometers were used to collect the specific forces of different carriers during the smooth motion phase, including the specific force of car, aircraft, waist and foot. The above specific force data and its partial amplification results are shown in Figure A3. Obviously, the specific force of the pedestrian is greater than that of the tradition carrier such as vehicle and the aircraft. This feature can increase the *f* of Equation (A9) and further increase the G-sensitivity error.

Referring to the performance indicators of Analog Devices Inc. (ADI)’s product-level MEMS gyroscopes, the G-sensitivity coefficient of consumer-level MEMS gyroscope is approximately 0.015°/s/g. At the same time, according to the data in Figure A3, combined with Equation (A9), the G-sensitivity error corresponding to each sampling point is obtained, as shown in Figure A4. The magnitude of the G-sensitivity error related to the foot is much larger than that of the relatively stable pedestrian waist, vehicle or plane, and it is between −0.08°/s~+0.1°/s. To further quantitatively analyze the problem, the attitude error caused by the G-sensitivity error of the foot in each gait cycle is numerically accumulated, and it can be found that the attitude error caused by the G-sensitivity error in each gait cycle is at least 0.6°. Considering that the pedestrian’s gait cycle is generally between 0.7 s and 1.3 s, it is concluded that the magnitude of the G-sensitivity error caused by the pedestrian’s foot motion characteristics has exceeded the drift of consumer-grade MEMS gyro.

Aiming at the G-sensitivity error of gyroscope, Fan [11] constructed the coefficients of G-sensitivity as a time-domain random walk model and estimated it with Kalman filter. Based on gyro output models and least-squares fitting methods, Xing [12] proposed a method for calibrating the coefficients of G-sensitivity. Most existing calibration methods for G-sensitivity error are based on the gravity environment, but the specific gravity caused by pedestrian movement far exceeds the specific gravity provided by the gravity environment. Therefore, the huge gap between the calibration environment and the actual environment inevitably brings errors. At the same time, MEMS gyroscopes used in the field of pedestrian navigation are usually low-cost, low-performance devices. Their high noise drift may even directly submerge the angular velocities caused by G-sensitivity error, which also affects the effect of calibration. Therefore, there is currently no effective method for calibrating the MEMS gyroscope G-sensitivity coefficient in pedestrian navigation application. Moreover, when the SINS is used for pedestrian navigation, it is limited by the fact that the G-sensitivity error is amplified.

In summary, for the SINS systems currently used in pedestrian navigation, the movement characteristics produced by pedestrians will increase the rate of angular velocity and the specific force, thereby amplifying the SINS cone error and the MEMS gyroscope G-sensitivity error. According to the coupling relationship of SINS error, the above two errors amplify the position error and heading error of the SINS further. Therefore, the pedestrian’s movement characteristics bring about problem that should not be neglected for SINS applied in pedestrian navigation.
